# Proteomics profiling asthma induced-lysine acetylation

**DOI:** 10.17179/excli2019-1508

**Published:** 2020-06-04

**Authors:** Xin-ming Su, Yuan Ren, Meng-lu Li, Shi-yao Bai, Na Yu, Ling-fei Kong, Jian Kang

**Affiliations:** 1 Department of Pulmonary and Critical Care Medicine, Institute of Respiratory Diseases, The First Affiliated Hospital of China Medical University, Shenyang, Liaoning 110001, China

**Keywords:** asthma, acetylation, acetylproteome, HDACi

## Abstract

Asthma is a chronic inflammatory disease that has been extensively studied for many years. However, finding a complete cure remains a significant challenge. Protein acetylation, especially histone acetylation, plays a significant role in the anti-asthma process. Histone deacetylation inhibitors (HDACi) have been shown to have a curative effect on asthma in clinical practice. An asthmatic mouse model was created by ovalbumin induction. Proteome and acetylproteome analysis were performed on lung tissues. HDACi were tested in the asthmatic mice. A total of 5346 proteins and 581 acetylation sites were identified, among which 154 proteins and 68 acetylation peptides were significantly altered by asthma. Many activated and deactivated processes, pathways, and protein groups were identified through bioinformatics analysis. Sequence motif preference analysis gave rise to a novel Kac-related core histone region, -KAXXK-, which was postulated as a key regulatory unit of histone acetylation. Asthma involves a variety of proteome dynamics and is controlled by protein lysine acetylation through the core motif -KAXXK-. These findings provide novel avenues to target and treat asthma.

## Introduction

Asthma is a common chronic inflammatory disease that leads to recurrent wheezing, shortness of breath, chest tightness, cough, and other associated symptoms. One of the main pathological features of the asthmatic state is hypoxia, followed by airway remodeling and inflammation (Barnes et al., 2005[5]; Ahmad et al., 2012[1]). Asthma is associated with a variety of inflammatory genes, such as cytokines, chemokines, inflammatory mediators, and related enzymes (Barnes and Karin, 1997[6]; Barnes and Adcock, 1998[4]). All of these genes differentially contribute to the activation of cell inflammation. Many of these genes are regulated by proinflammatory transcription factors including NF-κB and AP1, which activate and amplify inflammatory responses (Barnes and Adcock, 1998[4]).

Over the last few decades, several studies have established how inflammatory gene proteins, such as histone acetylation and methylation, are regulated (Ito et al., 2002[14]; Kwon et al., 2008[16]). In the case of allergic asthma, previous studies have shown that histone acetylases (HATs) activity will increase and specific cofactors will be recruited to HATs, thus amplifying histone acetylation, enhancing related gene transcription, and ultimately resulting in the cellular inflammation and other anti-asthma processes (Barnes et al., 2005[5]; Ogryzko et al., 1996[19]; Roth et al., 2001[22]). In contrast, histone deacetylase (HDAC) activities are reduced to keep the chromatin in a hyper-acetylated state, which is consistent with the recovery process. In recent years, asthma therapies targeting HATs and HDACs have been developed and clinical trials have shown that they have therapeutic effects on asthma (Hart et al., 2000[11]; Ito et al., 2000[13]; Barnes, 2009[3]).

To study the epigenetic targets of HDACi that have anti-tumor potential, we used an asthmatic mouse model to profile proteomic and acetylproteomic changes. We established the asthmatic mice model by induction with ovalbumin (OVA) and Al(OH)_3_ gel. A comprehensive analysis of acetylation-regulated processes that were induced by allergic asthma was performed. Protein sequence motif analysis revealed a key Kac motif that may be involved in OVA induced-asthma.

## Materials and Methods

### Generation of an asthmatic mouse model and drug treatment 

The mouse asthma model was generated as previously described (Temelkovski et al., 1998[28]; Lee et al., 2009[17]). Briefly, specific-pathogen-free, female BALB/C mice aged 6-8 weeks were treated with OVA (20 μg/0.2 ml) and Al(OH)_3_ gel (2 mg) on days 1, 8, and 15 to induce an allergic asthmatic response. In the 8 weeks after sensitization, an ultrasonic atomization device was used 3 times per week to perform OVA atomization stimulation (3 ml/min, 20 mg/ml) for 30 minutes each time. For the control group, mice were treated with normal saline (0.2 mL) and Al(OH)_3_ gel (2 mg) on days 1, 8, and 15. In the 8 weeks after the sensitization, the same ultrasonic atomization treatment used for the OVA-treated mice was given to the control group.

Dexamethasone (2.0 mg/kg) (Zhuo Feng Pharmaceutical Co., Ltd., Zhengzhou, China) (Fu et al., 2014[9]), Tubastatin A Hcl (TSA, 0.5 mg/kg) (Wang et al., 2014[29]), and PCI-34051 (0.5 mg/kg) were administered via intraperitoneal injection for 30 min before excitation. In the control group, normal saline was used to replace OVA. All HDAC inhibitors mentioned above were purchased from Selleckchem, Houston, TX, USA.

### Proteomic and acetylproteomic analysis

The workflow of quantitative proteomic and acetylproteomic analysis is provided in Supplementary Figure 1. In brief, for the proteomic strategy, mouse lung tissues were harvested and ground into powder using liquid nitrogen and followed by protein extraction. After trypsin digestion and TMT (Tandem Mass Tag) labeling, peptide samples from both the control and asthma mice were mixed at a 1:1 ratio (w/w). Subsequently, HPLC fractionation was used to fractionate the peptides. The whole sample was separated into 18 fractions and sent to LC-MS/MS for quantification and bioinformatics analysis was performed. Also, other methods used in this study are included in the Supplementary Material.

## Results

### Identification of asthma associated factors using proteomics analysis

To identify changes in the proteins expression and signaling pathways associated with the asthmatic response, we carried out proteomic analyses using an OVA-induced allergic asthma mouse model (details in Supplementary Material).

**Figure 1:**
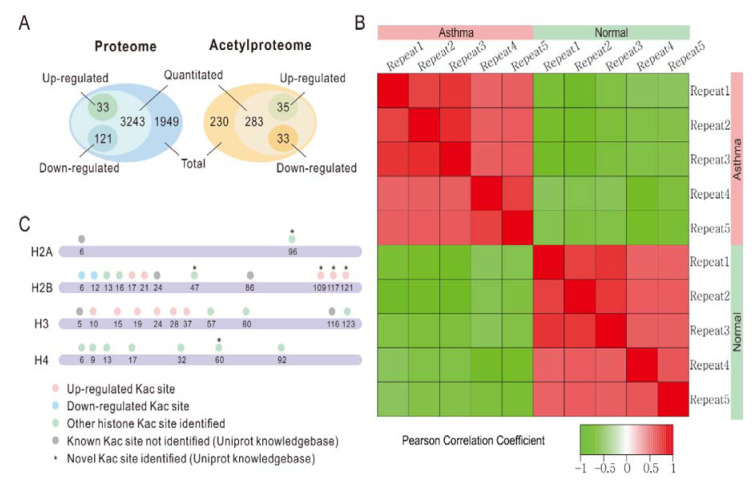
Identification of candidate proteins involved in the asthmatic response. A. Venn diagram of proteomic and acetylproteomic data. B. Pearson correlation coefficient of each replicate. C. Kac identification coverage of core histones. Dots stand for Kac sites. Orange, up-regulated Kac site. Blue, down-regulated Kac site. Green, other Kac site identified in our study. Gray, known Kac site in UniProt knowledge base but not identified in our data. * indicates a novel Kac site according to the UniProt knowledgebase.

**Figure 2:**
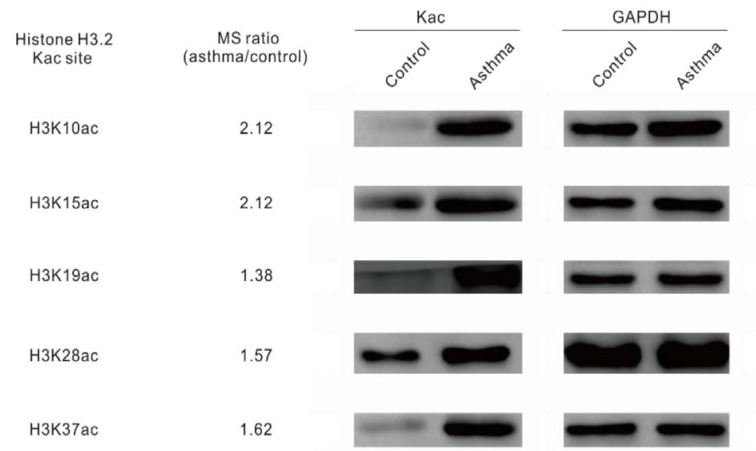
Western blot of histone acetylation on Kac sites. Immunoblots of separated protein samples from the lung tissues of mouse asthma models with Kac site specific antibodies. GAPDH was used as the loading control.

Overall, we identified 5346 proteins in the lung tissues of normal and asthmatic mice, and 3397 of them were quantitatively analyzed by proteomic analysis (Figure 1A(Fig. 1), Supplementary Table 1 and 2). The Pearson correlation coefficient showed good repeatability of our data (Figure 1B(Fig. 1)). We used a 1.3 fold (asthmatic group/control group) criteria for selection and identified 33 up-regulated proteins and 121 down-regulated proteins in the asthmatic group. In the acetylproteomic study, 581 lysine acetylation sites in 335 protein groups were identified, among which, 351 sites in 215 proteins were quantified. When setting the alteration of the Kac site to more than 1.2 fold (asthmatic group/control group) as the screen criteria, 35 up-regulated sites and 33 down-regulated sites were obtained (Figure 1A(Fig. 1)). Regulation of acetylation was frequently detected in histone proteins. Overall, 15 out of 39 (*ca. *38.5 %) Kac peptides had quantitative changes to histone acetylation sites (including the same Kac site in different histone variants, Table 1(Tab. 1)), among which, only two Kac sites were significantly down-regulated (in H2B). Interestingly, all regulated lysine acetylation occurred in histone-2B (H2B) and H3 (9 out of 19 Kac-site-containing peptides were changed in H2B, and 6 out of 10 Kac peptides were changed in H3, ~51.7 % acetylation sites changed, Table 1(Tab. 1)), but no acetylation regulation was observed in H1, H2A, and H4 (10 quantitative acetylation sites in total). Therefore, suggesting a potential role of acetylation up-regulation in H2A and H3 during asthma-related gene regulation. According to UniProt, when combining the same Kac sites derived from different histone variants as a single site for non-redundant site analysis, we identified most known Kac sites in core histones, and only 5 Kac sites were missed (Figure 1C(Fig. 1)). Furthermore, 6 novel histone Kac sites were identified for the first time, including K96 in H2A, K47, K109, K117, and K121 in H2B and K60 in H4. This indicates a very good coverage of the core histone Kac sites and provides evidence that the histone Kac analysis based on our data is systematic and comprehensive. We validated the alteration of 5 specific Kac sites in histone (Figure 2(Fig. 2)). The Western blot data showed the same alteration trend as our mass spectrometry (MS) data and indicated that the fold change of peptides detected by MS might be lower than what was found in this study. Therefore, we selected 1.2 fold as the criteria of a significant change.

### Functional cluster and enrichment analysis of proteome and acetylproteome

To analyze the role of protein acetylation, we performed a Gene Ontology (GO) analysis to cluster the regulated and acetylated proteins. Biological process, molecular function, and cellular-component-based GO analyses were performed and only clusters containing more than 3 proteins were chosen for further analysis.

Since only 68 differentially altered acetylation sites were detected in our study, which may not fully reflect all the processes and changes involved in asthma, we divided all quantitated acetylation proteins into four quantiles according to their quantitation ratio. Q1 the 25 % in the lowest-quartile are the down-regulated proteins and the highest-quartile (Q4) are the upregulated proteins, respectively. Q2 and Q3 refer to 25 %-50 % and 50 %-75 % ratio proteins. A single, regulated acetylation protein is only a dot in the pathway involved. Therefore, it cannot reflect the whole regulation trend, but quantile analysis can cover more proteins with the same or similar function. Thus, it reveals the corresponding regulation in a more persuasive and realistic pattern. We used this method to analyze the proteomic and acetylproteomic cluster enrichment of biological process, molecular function, cellular component, KEGG pathway, and protein domain based on quantile classification. Many clusters that were identified have been reported in previous studies (Zhang et al., 2009[32]; Quesada Calvo et al., 2011[21]); however, some novel clusters were also connected to the development of asthma development at the molecular level.

In Q1 of regulated proteins, molecular function clusters such as oxidoreductase/antioxidant activity and peptidase related activity were enriched. In the biological process, protein clusters such as responses, metabolic/catabolic processes, reactive oxygen species (ROS)/peroxide/superoxide related processes, and ribosomal related processes were enriched. This suggests that certain responses, perhaps those related to antigen stimulation, were suppressed to reduce the allergic process. Superoxide and ROS processes were down-regulated possibly because of low oxygen concentrations. Cellular components including membrane-related lumen/vesicle, respiratory chain, and ribosomal-related components were also enriched in Q1 proteins, which indicates decreased secretion activity and aerobic respiration processes. Protein clusters in Q4 were different from those in Q1. Molecular functions including RNA-cap binding were enriched, revealing mRNA transcription processes in asthma was highly active. In biological processes and cellular components, adhesion- and junction-related proteins were enriched, which may be related to changes in the biological behavior of airway epithelial cells and smooth muscle cells in asthma. In addition, laminin-related proteins were enriched in the protein domain cluster, which is in agreement with the previous finding that the extracellular matrix was increased in asthmatic patients (Singh et al., 2012[26]; Yick et al., 2012[31]).

### Motif analysis of acetylation sites in asthmatic mouse lungs

**Figure 3:**
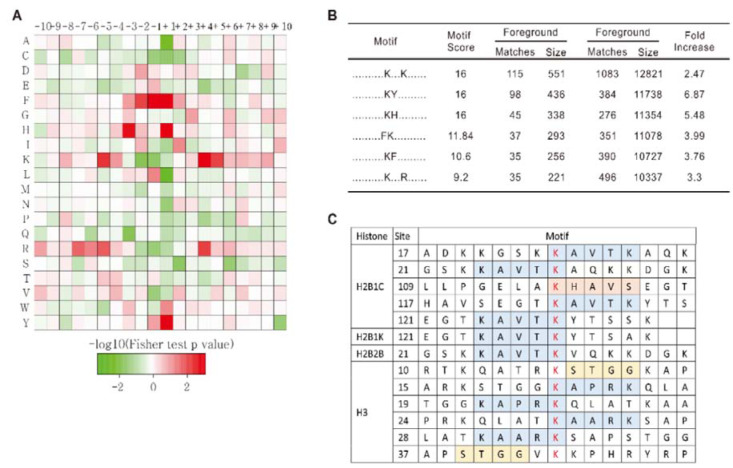
Motif analysis of acetylproteomic data. A. Sequence distribution preference of Kac sites. Red indicates high frequencies and green indicates low frequencies of specific amino acids. B. The most frequent motif sequences identified in our data. C. All motif sequences of up-regulated Kac sites identified in our study revealed a strong histone Kac motif preference in mouse asthma models.

Studies of functional regulation induced by Kac alteration is important to understand the activated processes and pathways in the asthmatic response. Clinical practice has verified that HDACi play a significant role in asthma treatment. To find the active corresponding HATs or HDACs under asthmatic conditions, we used a motif analysis to conclude the principal structure or sequence trend of amino acids distributed around Kac sites (Figure 3(Fig. 3)). The analysis of amino acid frequencies shows that F, H, and Y residues are more likely to exist around the Kac site. Furthermore, A, L, C, and S residues are more likely to be absent. In addition, K is strongly associated with positions -5, +4, and +5 around the Kac site and is absent at positions -1 and -2 site around lysine Kac sites. Moreover, amino acid frequency around R is similar to that around K (Figure 3A(Fig. 3)). The analysis of motif sequences (Figure 3B(Fig. 3)) found that the sequences XXXXXXXXXKXXXKXXXXXX (one X stands for one amino acid), XXXXXXXXXXKYXXXXXXXXX, and XXXXXXXXXXKHXXXXXXXXX had the highest score, which indicates that these amino acids existed more frequently. In addition, XXXXXXXXXFKXXXXXXXXXX, XXXXXXXXXXKFXXXXXXXXX, and XXXXXXXXXXKXXXRXXXXXX were also highly frequent sequences. This result suggests that these amino acid sequences were probably the preferred amino acid sequences for HATs and HDACs in the selection of their substrates. After analyzing histone lysine acetylation, we found a strong structural preference for up-regulated Kac sites (Figure 3C(Fig. 3)). In 2B, most up-regulated Kac sites were located either side of the -KAVTK- motif. In H3, up-regulated Kac sites were located either side of the -KAPRK- or -KAARK- motif. Overall, we found that the up-regulated expression of related genes in asthma was probably related to the increased acetylation of the amino acid sequence -KAXXK- on histone.

### Histone Kac site variance determines asthma treatment

Our data were compared to a previous study (Scholz et al., 2015[25]) that established the influence of HDACi on histone Kac sites. This provided functional validation of our data and revealed the relationship between HDACi, HDAC, and histone Kac sites (Table 2(Tab. 2); Reference in Table 2: Scholz et al., 2015[25]).

Over the past few years, many studies have investigated HDAC deactivation induced by HDACi to develop a treatment for allergic asthma. However, the specific histone Kac sites that are affected by HDACi treatment and also the naive target for HDAC-based cures remains unknown. Fortunately, Chunaram's group used a meta-analysis to reveal the effect of 19 common HDACi on acetylation sites of histones, which provided an excellent database for our current analysis (Scholz et al., 2015[25]). We defined a value of histone Kac site variance, to assess the difference between the up-regulated Kac sites in our data and Kac sites influenced by HDACi. For example, in our study, TSA influenced most up-regulated Kac sites except from two sites; therefore, the histone Kac site variance was 2. Based on this setting, we hypothesized that HDACi with less variance were more likely to enhance the recovery processes in allergic asthma. After a summary of the current information or application of mentioned HDACi listed in Table 2(Tab. 2), we get the following novel findings: (1) all HDACi with variance of 2 or less were involved in asthma-related processes (Yamamoto et al., 1996[30]; Choi et al., 2005[8]; Han et al., 2007[10]; Banerjee et al., 2012[2]; Royce et al., 2012[23]; Royce and Karagiannis, 2012[24]; Morschhauser et al., 2015[18]). Moreover, all of these HDACi shared the same target with HDAC 1-3, 2) HDACi with variance between 3 and 5 had a target preference that only HDACi with HDAC 1-3 inhibitor activity are associated with the treatment of asthma (Scholz et al., 2015[25]; Royce and Karagiannis, 2012[24]; Prince and Prince, 2009[20]), and 3) with increased variance, HDACi regain their asthma-treating ability regardless of their targets (Kim et al., 2010[15]; Sutcliffe et al., 2012[27]; Bosnar et al., 2013[7]). These findings suggest that HDAC 1-3 are the key regulatory groups in asthmatic processes.

### Reduced asthmatic response by acetylation inhibitors

**Figure 4:**
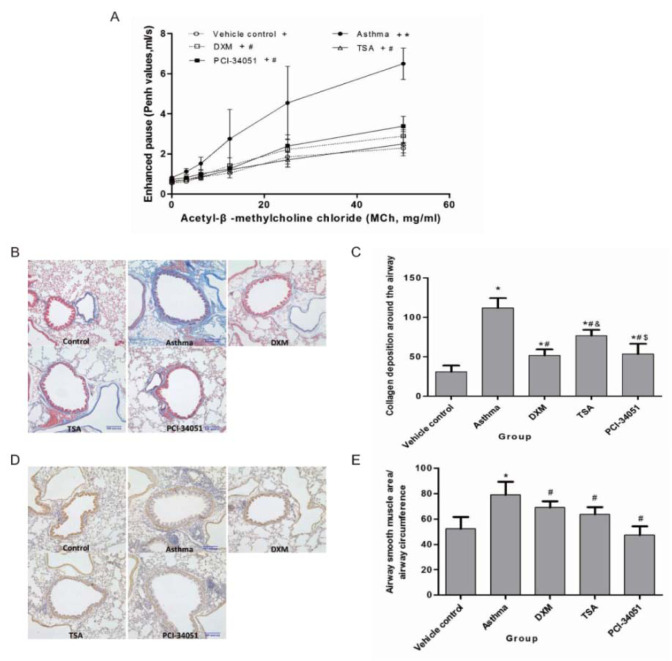
Validation of HDACi in airway resistance, collagen deposition in the sub-epithelium, and alpha-SMA alteration. A. The airway resistance assay. Comparison of enhanced pause (Penh values) upon stimulation of various concentrations of methacholine (MCh). Data are presented as mean ± SD at different concentrations of MCh for each group (n=8). *p*<0.05 indicated the Penh values increased with increasing concentrations of MCh for each group (all *p*-values <0.001). *p*<0.05, compared with the normal control group (marked as *), or the asthma group (marked as #). B & C. The collagen deposition assay and its statistical significance. The ratio of collagen deposition area around the airway and the circumference of the airway was detected by Masson staining. D & E. The alpha-SMA alteration assay and its statistical significance. Significant differences were identified using a one-way ANOVA. In pane C and E, *p*<0.05 indicates a significant difference from the control (*), asthmatic (#), or DXM groups (&).

**Figure 5:**
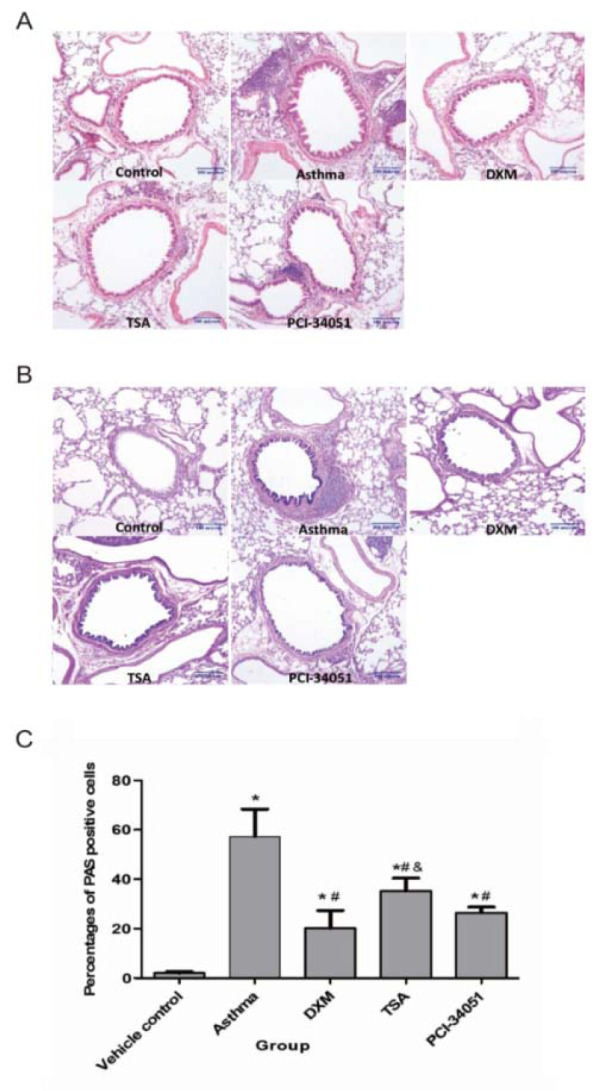
Anti-inflammatory effect of HDACi in t asthmatic lung tissues. A. HE staining shows infiltration of the inflammatory cells. B & C, the PAS staining assay and its statistical significance. Percentages of PAS positive cells in airway epithelium in each group are presented here as means ± SD (n=6). *p*<0.05 indicates a significant difference from the control (*), asthmatic (#), or the DXM groups (&).

To validate our assumption, we selected two HDACi, TSA and PCI-34051, which are commercial drugs for the treatment of asthma. According to our comparison, TSA only has a histone Kac site variance of 2 and PCI-34051 has more than 6. The former is proposed to promote the recovery process through regulation of a specific histone motif (-KAXXK-). Furthermore, dexamethasone (DXM) was used as a positive control to indicate the efficacy of the selected HDACi. First, a series of pathological features including airway resistance, collagen deposition under the epithelium, and alpha-SMA (α-smooth muscle actin) alteration were examined in five groups consisting of the control group, the asthma group, DXM-treated asthma group, TSA-treated asthma group, and PCI-34051-treated asthma group (Figure 4(Fig. 4)). The results showed airway resistance was increased in the asthma group but it was reduced by DXM, TSA, and PCI-34051 treatment. Among these three drugs, TSA appeared to have better efficacy than DXM and PCI-34051; however, this was not statistically significant. Similarly, collagen deposition and alpha-SMA staining were induced by asthma and decreased by drug treatments (Figure 4(Fig. 4) and 5(Fig. 5)). 

**Figure 6:**
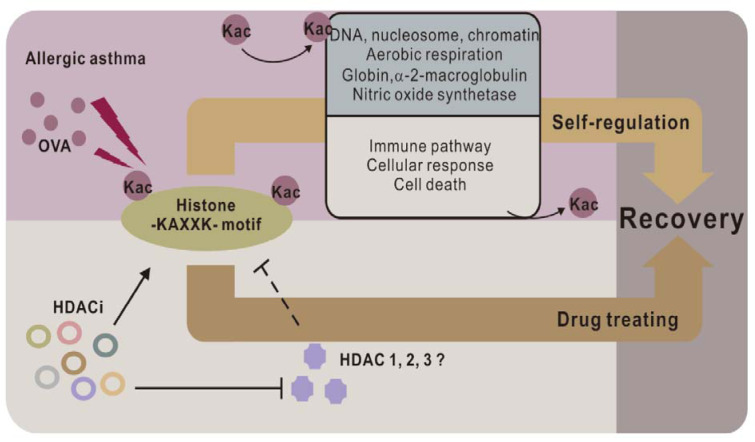
-KAXXK- as a key Kac motif in anti-asthmatic processes. Through altering gene expression, acetylation of -KAXXK- reduces the activity of the immune system and activates anti-asthmatic process. Current HDACi drugs targeting the histone -KAXXK- motif may also function through a similar mechanism.

Finally, we examined the inflammation features using cell infiltration and Periodic acid-Schiff (PAS) staining (Figure 5(Fig. 5)). PAS stains glycoproteins and so can be used to discriminate between different forms of interstitial lung disease (Hauber and Zabel, 2009[12]). As shown in Figure 6(Fig. 6), inflammation was induced in asthmatic mice and all drug treatments relieved this pathological response.

## Conclusion

In this study, we integrated Kac site enrichment, and an MS-based quantitative proteomic strategy to study the proteomic and acetylproteomic changes in the asthmatic lung tissues of mice. GO analysis and corresponding enrichment analysis were performed to > cluster the activated and deactivated protein groups, processes, pathways, to conclude dynamic change principles, and to reveal the relationships between clinical symptoms and molecular changes. Furthermore, a motif sequence preference of Kac sites in asthmatic lungs was performed through motif analysis, and a novel Kac-related core histone motif -KAXXK- was discovered as the target against asthma. Thus, this suggests that -KAXXK- is the key motif and its acetylation is responsible for anti-asthmatic processes and controls the expression of the corresponding genes. The corresponding genes are involved in subsequent anti-asthmatic processes and recovery from the disease.

Our acetylproteomic study suggested that most up-regulated histone Kac sites occurred in the -KAXXK- domain. Many known HDACi drugs for asthma treatment can increase histone Kac sites or acetylation of the -KAXXK- domain. Therefore, one of the anti-asthmatic mechanisms in the lungs of mouse models might be the acetylation of the histone -KAXXK- domain, which can also be achieved by deactivation of HDACs 1-3.

In addition to identifying biomarkers, we also identified Kac-induced activation of gene transcription and protein-expression-related clusters including nucleosome, chromatin-involved biological processes (BP, Q4), cellular components such as chromosome; nucleus (CC, Q4), and histone-related structures (Q4). These changes are involved in anti-asthmatic responses through the regulation of protein expression regulation. Moreover, cellular component organization (BP, Q4) and protein dimerization (MF, Q4) suggest that the organism strengthens the component and protein organization under asthmatic conditions to enhance many corresponding processes. As a result, the process of cell death (BP, Q1) was inactivated, which suggests that cells were recovering from asthma.

## Acknowledgement

None.

## Funding

This study was supported by grants from National Natural Science Foundation of China (No. 81770021) and Natural Science Foundation of Liaoning Province (No. 201701664).

## Conflict of interest

The authors declare that they have no conflict of interest.

## Authors’ contribution

Su XM, Kang J conceived and designed the experiments; Ren Y, Li ML, Yu N and Su XM performed the experiments; Su XM, Bai SY and Ren Y analyzed the data; Bai SY and Kang J contributed reagents/materials/analysis tools; Su XM prepared the manuscript. All authors have read and approved the final manuscript.

## Supplementary Material

Supplementary material

Supplementary tables

## Figures and Tables

**Table 1:**
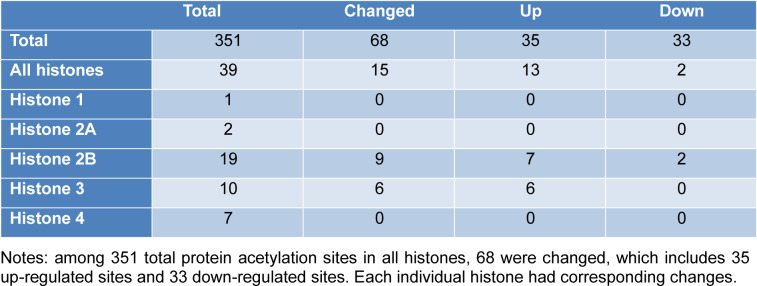
Table 1: Summary of the protein acetylation sites

**Table 2:**
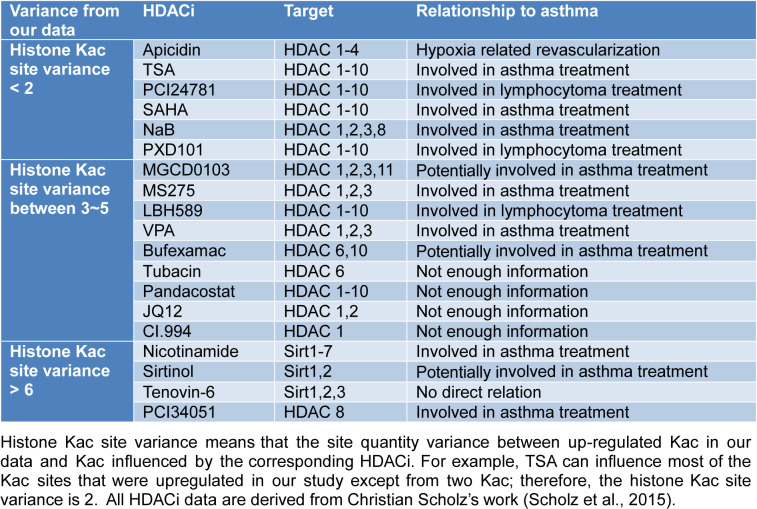
Table 2: Histone Kac site variance between previous work and our data
