# Supplementary material for: Proteomics profiling asthma induced-lysine acetylation
Source: EXCLI J. 2020 Jun 4;19:734–44. doi: 10.17179/excli2019-1508 (PMC7332788; doi:10.17179/excli2019-1508)
Supplement: Supplementary material [file EXCLI-19-734-s-001.pdf]

EXCLI Journal 2020;19:734-744 – ISSN 1611-2156
Received: March 09, 2020, accepted: May 25, 2020, published: June 04, 2020

Supplementary material to:

PROTEOMICS PROFILING OF ASTHMA INDUCED-LYSINE ACETYLATION

Xin-ming Su*, Yuan Ren, Meng-lu Li, Shi-yao Bai, Na Yu, Ling-fei Kong, Jian Kang

Department of Pulmonary and Critical Care Medicine, Institute of Respiratory Diseases, The First Affiliated Hospital of China Medical University, Shenyang, Liaoning 110001, China

* **Corresponding author:** Dr. Xin-ming Su, Department of Pulmonary and Critical Care Medicine, Institute of Respiratory Diseases, The First Affiliated Hospital of China Medical University, No. 155 North Nanjing Street, Shenyang, Liaoning 110001, China, Tel: +86-024-961200; Fax: +86-024-961200; E-mail: xinming_med@126.com

http://dx.doi.org/10.17179/excli2019-1082

This is an Open Access article distributed under the terms of the Creative Commons Attribution License (http://creativecommons.org/licenses/by/4.0/).

SUPPLEMENTARY METHODS

1. Western blot for protein expression

30-50 mg lung tissues were homogenized and lysed with RIPA buffer. Protein samples were separated with 15 % SDS-PAGE, transferred into a PVDF membrane, and blocked with the non-fat milk. After PBST washing, the membrane was further incubated with the secondary antibody. Finally, the blots were developed in the ECL chemi-luminescent substrate reagent, and the blot images were captured with the UVP digital imaging system.

All polyclonal antibodies against H3K9ac, H3K14ac, H3K18ac, H3K27ac and H3K36ac were purchased from Jingjie Biotech Inc (Hangzhou, China). HRP-conjugated secondary antibody was ordered from Zhongshan Golden Bridge Inc. (Cat#ZB-5301).

2. Methacholine-induced AHR (Airway Hyper Reactivity)

24 h after the last OVA challenge, AHR was measured using a mouse non-invasive whole body plethysmography (EMKA, French). The mice were placed in a whole-body plethysmography chamber with a ventilation of 0.5 Lmp/min. After baseline airway resistance was recorded, increasing concentrations of methacholine (3.125 mg/ml, 6.25 mg/ml, 12.5 mg/ml, 25 mg/ml, 50 mg/ml) was delivered by aerosol generator (EMKA, French) and airway resistance changed from baseline level was measured for three minutes. Penh (enhanced pause) was taken to express changes in murine airway function.

3. AB-PAS staining for mucus hypersecretion

The paraffin-embedded tissue section was de-waxed, washed in 3 % acetic acid, and stained with Alcian blue (Leagene, Beijing, China). Next, the tissue section was washed in distilled water, stained with 0.5 % periodic acid, washed again in distilled water, 70 % alcohol, and

stained with Schiff's reagent (15 min). Subsequently, wash the sample section in running tap water (10 min) and stain the section with hematoxylin. Finally, the sections were cleaned in running tap water, dehydrated with a graded alcohol series, vitrificated in xylene, and mounted in resinous medium. The proliferation of goblet cells in the airway was observed under a light microscope.

4. Masson's trichrome staining for collagen deposition assay

The tissue sections were de-waxed and stained with Masson's composite staining solution (Fuzhou Maixin Bio, Fujian, China) for 5 min. Subsequently, the sections were washed in 0.2 % acetic acid, stained with 5 % tungsten molybdate (5-10 min), washed in 0.2 % acetic acid twice, stained with aniline blue (5 min), dehydrated in a graded alcohol series, vitrificated in xylene and mounted in the resinous medium. The subepithelial collagen deposition was observed under a light microscope.

5. alpha-SMA immunohistochemistry staining

De-waxed paraffin sections were treated with in 3 % H_2O_2 at room temperature for 10 min to inactivate the endogenous peroxidase, followed by antigen retrieval treatment with citric acid buffer (pH 6.0). After blocked with 15 % goat serum for 30 min, tissue sections were incubated with alpha-SMA antibody (Abcam, 1:1000) at 4 ºC overnight, washed with PBS for 3 times (5 min/time) and incubated with a second antibody (1:500) at room temperature for 60 min. The staining signals were developed with the DAB reagent for 5-10 min. Finally, the sections were washed with H_2O for 10 min, re-stained with hematoxylin as mentioned above.

6. Quantitation and analysis of proteomic and acetylproteomic data

Workflow of quantitative proteomic and acetylproteomic study is shown in Supplementary Figure 1. For proteomic strategy, lungs from each mouse group (asthmatic mouse model building method, see Material and Method section) were taken out and grinded into powder in liquid nitrogen followed with protein extraction. After trypsin digestion, each group was divided into five individual groups as replicates followed with TMT labeling. Then, peptide samples were mixed at w/w=1:1. Then HPLC fractionation was applied to fractionate the peptides, the whole sample was separated into 18 fractions and then sent to LC-MS/MS. At last, quantification information was acquired and bioinformatics analysis was performed. Acetylproteomic strategy was similar to proteomic strategy. When tryptic peptides were collected, sample group and control group were mixed at w/w=1:1. Acetylpeptides were enriched through acetyl lysine antibody. After enrichment, acetylpeptide samples were sent for LC-MS/MS analysis and the following bioinformatics analysis.

Before data analysis, we first checked the mass error of all identified peptides. Mass error dots distribute around zero and most of them are less than 0.02 Da which reveals that the mass accuracy of our MS data (Supplementary Figure 1). Second, distribution of peptide length is between 8 and 20, this indicates a good property of tryptic peptides.

7. Protein extraction and trypsin digestion

Lungs of the mice were cut into pieces after taken out from the bodies, the followed liquid nitrogen grinding was performed and the protein powder was collected into a 1.5 ml tube. 2 ml lysis buffer (8 M urea, 3 µM TSA, 50 mM NAM, 65 mM DTT, 1 % protease inhibitor cocktail, 50 mM Tris-HCl), with 1 % Triton X-100, 2 mM EDTA, pH 7.5 was used to dissolve proteins followed with a 6 min sonication in ice water. Added 1/6 sample volume TCA into sample drop by drop. After 2 h in 4 °C, the protein sample was centrifuged in 5,000 g for 3 min and the supernatant was discarded. Pre-cold acetone was used to wash sample for 3 times, re-suspended

for 10 min each time and then the sample was air-dried. After that, proteins were re-dissolved in 1 ml 8 M urea, then centrifuged at 12,000 g for 3 min and the proteins were dissolved in supernatant. A final concentration of 10 mM DTT was added into the sample in 37 °C for 1 h followed with a final concentration of 20 mM IAA treatment for 45 min in darkness for sample reduction and alkylation. After that, ddH_2O was used to attenuate sample until concentration of urea was below 2 M and TEAB was above 100 mM. Added trypsin at a ratio of 1:50(w/w) to digest proteins overnight, the second digestion was performed at a ratio of 1:100(w/w) for 4 h.

8. TMT/iTRAQ labeling

Digested peptides were desalted with Strata X C18 (Phenomenex) and then vacuum freeze-dried. The desalted peptides were dissolved with 0.5 M TEAB and the peptides were labeled according to Kit's instruction. In one TMT kit, each labeling reagent (for 100 µg peptide) was dissolved with 80 µg ACN and mixed with corresponding peptide sample in room temperature for 2 h. The labeled peptides were desalted again and then vacuum freeze-dried for following treatment. The labeling strategy was listed as followed, A stands for asthmatic mice, B stands for healthy mice, a total of 5 replicates for each group was labeled so we needed two kits for labeling. In Kit 1, TMT-126 for A1, TMT-127 for A2, TMT-128 for A3, TMT-129 for B1, TMT-130 for B2, TMT-131 for B3. In Kit 2, TMT-126 for A4, TMT-127 for A5, TMT-129 for B4, TMT-130 for B5.

9. HPLC fractionation

High pH reverse HPLC with an Agilent 300 Extend C18 column (5 µm particles, 4.6 mm ID, 250 mm length) was used for fractionation. Buffer A (pH 10) comprises 98 % H_2O, 2 % ACN, and 10 mM ammonium formate. Buffer B (pH 10) comprises 10 % H_2O, 90 % ACN, and 10 mM ammonium formate. When the fraction gradient was running, buffer B was increasing from an initial concentration of 2 % to 5 % in the first 5 min, then increased to 8 % in 5 min, to 18 % in 25 min, to 32 % in 22 min, to 95 % in 2 min, stayed in 95 % for 4 min, and at last back to 5 % in 4 min. Flow rate was set to 300 nl/min. Elution from 16 min to 74 min was collected into 58 sub-fractions and selected every other 18 sub-fractions and finally combined to 18 fractions (for example, sub-fractions 1, 19, 37, 55 are combined into one fraction).The final fractions were vacuum freeze-dried for the following treatment.

10. Acetylation peptides enrichment

Peptides were dissolved in IP buffer (100 mM NaCl, 1 mM EDTA, 50 mM Tris-HCl, 0.5 % NP-40, pH 8.0) and transferred into prepared acetyl beads (PTM Biolabs), then mild shaked in 4 °C and incubated overnight. After incubation, beads were washed for four times with IP buffer and twice with ddH_2O and finally eluted for three times using 0.1 % TFA. Elution was collected, vacuum freeze-dried, desalted with C18 ZipTips (Millipore) and a second time vacuum freeze-dried for LC-MS/MS analysis.

11. LC-MS/MS analysis

Peptides were dissolved in 0.1 % formic acid, loaded into a reverse pre-column (Acclaim PepMap 100, Thermo Scientific), and then a reverse analytical column (Acclaim PepMap RSLC, Thermo Scientific). EASY-nLC 1000 ultra-performance liquid system was applied to sample fraction. Buffer A comprises 0.1 % formic acid, 2 % ACN. Buffer B comprises 0.1 % formic acid, 98 % ACN. When the fraction gradient was running, buffer B was increasing from an initial concentration of 7 % to 20 % in the first 24 min, then increased to 35 % in 8 min, to 80 % in 5 min, and at last stayed in 80 % for 3 min. Flow rate was set to 300 nl/min.

EXCLI Journal 2020;19:734-744 – ISSN 1611-2156
Received: March 09, 2020, accepted: May 25, 2020, published: June 04, 2020

Samples fractioned by LC were analyzed by Thermo Scientific TM Q ExactiveTM Plus. Peptides were sent to NSI source followed by MS/MS analysis. The electrospray voltage was set to 2.0 kV, intact peptide and its fragment ions were analyzed by Orbitrap. MS scan range was set to 350 to 1800, and fixed first mass was set as 100 m/z. Resolutions for intact peptide and fragment ion were set to 70,000 and 17,500, respectively. A data-dependent procedure that alternated between one MS scan followed by 20 MS/MS scans was applied for the top 20 precursor ions above a threshold ion count of 5E3 in the MS survey scan with 15.0 s dynamic exclusion.

12. Database search

The MS/MS data was searched by MaxQuant with integrated Andromeda search engine (v.1.4.1.2). Spectra were searched against UniProt_Mus musculus database concatenated with reverse decoy database. Trypsin/P was set as the cleavage enzyme, missed cleavage was set to 4, modifications per peptide were set to 5, and charges were set to 5. The mass tolerance was set to 10 ppm for precursor ions and 0.02 Da for fragment ions. Carbamidomethylation on Cys was set as fixed modification and oxidation on Met, acetylation on Lysand on protein N-terminal were specified as variable modifications. Minimum peptide length was set at 7. False discovery rate (FDR) thresholds for protein, peptide were specified at 1 %. The site localization probability was set as > 0.75.

13. Bioinformatics analysis

Gene Ontology (GO) annotation data was derived from UniProt-GOA database (http://www.ebi.ac.uk/GOA/). GO clusters were manually performed by matching identified proteins to database proteins and the corresponding clusters. For enrichment analysis, Functional Annotation Tool of DAVID Bioinformatics Resources 6.7 was introduced for identifying enriched GO against background Homo sapiens GO. The enrichment of each cluster was tested by two-tailed Fisher's exact test. The GO with a corrected p-value < 0.05 was considered significant. Motif-x was employed to analyze the amino acid sequences around the specific Kac site (10 amino acids up- or down-stream of the Kac site) in all protein sequences. All the protein sequences in database were used as the background database. Other parameters were set as default.

14. Lung tissue HE staining

Paraffin sections were de-waxed, stained with hematoxylin for 10 min, washed with H_2O for 5 min and differentiated for 3 s at room temperature with ethanol in HCl. After being washed with H_2O for 30 min until the blue color came back, the sections were stained with eosin for 2 min, washed gently with H_2O, dehydrated with in alcohol gradient, transparentized with dimethylbenzene and mounted with neutral balsam. The images were taken under optical microscopy.

EXCLI Journal 2020;19:734-744 – ISSN 1611-2156
Received: March 09, 2020, accepted: May 25, 2020, published: June 04, 2020

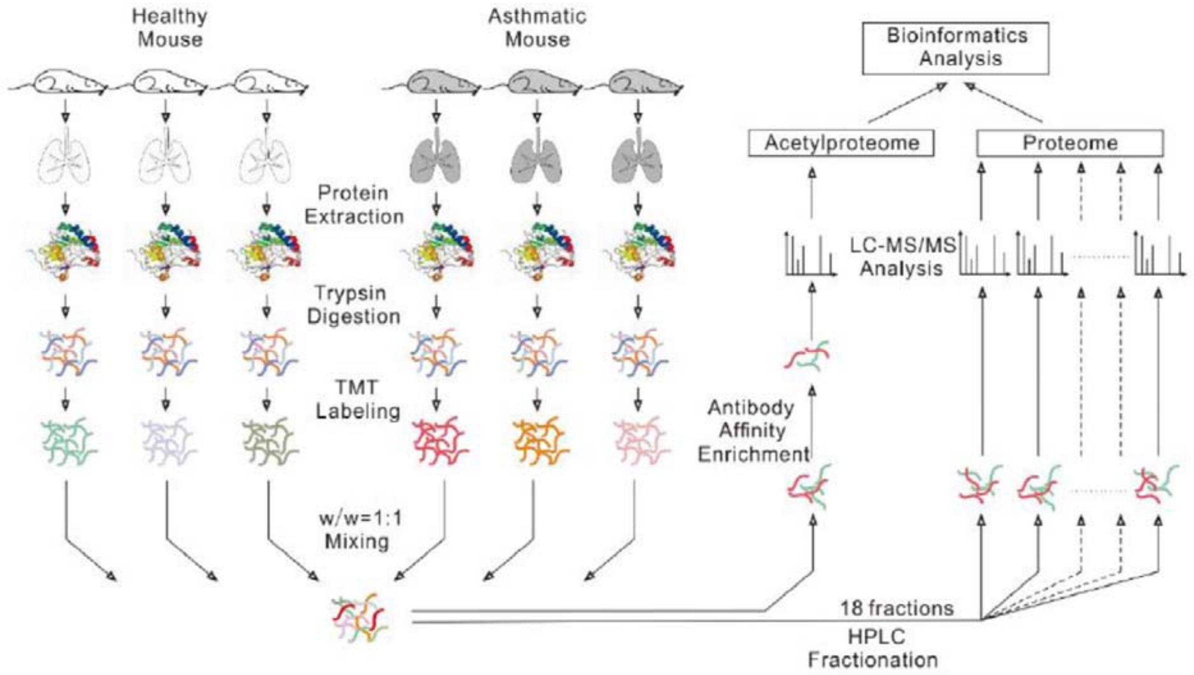

Supplementary Figure 1: Work-flow of quantitative proteomic and acetylproteomic study. Lung tissues from OVA/Al(OH)3 induced (asthmatic) and healthy mice were subject to protein extraction, trypsin digestion, TMT labeling, and mixing at the ratio of 1:1 (w/w). For the proteomic study, HPLC fractionation was performed to obtain 19 less complex fractions. For the acetylproteomic study, an anti-Kac antibody was used. Peptide samples were analyzed with mass spectrometry for sequencing.
